# Characterization, Cure Rates and Associated Risks of Clinical Mastitis in Northern Germany

**DOI:** 10.3390/vetsci7040170

**Published:** 2020-11-03

**Authors:** Anne Schmenger, Volker Krömker

**Affiliations:** 1 Department of Bioprocess Engineering, Microbiology, Faculty II, Hannover University of Applied Sciences and Arts, D-30453 Hannover, Germany; anne.schmenger@hs-hannover.de; 2 Department of Veterinary and Animal Sciences, Section for Production, Nutrition and Health, Faculty of Health and Medical Sciences, University of Copenhagen, DK-1870 Frederiksberg C, Denmark

**Keywords:** bovine mastitis, risk factors, severity score, mastitis-causing pathogen, recurrence rate, *Streptococcus uberis*

## Abstract

The control of clinical mastitis on dairy farms is an essential part of animal health management. Knowledge of the causative microorganisms, the cure rates achievable in the field and essential associated factors are crucial for proper control. The objectives of the present study were to characterize clinical mastitis cases in Germany and to analyze factors influencing cure rates and the recurrence rate. Milk samples of every clinical mastitis case occurring on 12 participating farms were examined cytomicrobiologically. Post-treatment quarter samples were taken after 14 and 21 days. Treatments were performed according to existing farm protocols. Of 2883 clinical mastitis cases, the most prevalent pathogens were *Streptococcus* (*S*.) *uberis* (20.2%) and coliforms (11.6%). In 35% of the milk samples, no bacteriological growth was detected. The overall bacteriological cure rate was 73.3%, while the cytological cure rate was 22.3%, the full cure rate 21.4% and the recurrence rate 18.8%. Regarding the pathogen distribution of severe mastitis, coliform bacteria were detected in 30.5% of the cases, whereas *S. uberis* was detected in 26.5% thereof. The results show that severe mastitis is caused almost as frequently by Gram-positive as by Gram-negative microorganisms. The low cytological cure rates show that the therapy needs to be further developed with regard to calming the inflammation. The obtained data can be very helpful in assessing internal mastitis scenarios and the effect of measures and therapies.

## 1. Introduction

Although clinical mastitis has been the focus of numerous research studies, there is a lack of basic up-to-date data on the clinical cases actually occurring in Germany. Likewise, to date, there are no published articles on currently achieved cure rates with standard treatment protocols and related animal risk factors, which are decisive for the outcome. It is essential to regularly describe clinical mastitis cases in order to know which clinical manifestation can be caused by which pathogen, as the dominant mastitis-causing organisms are constantly shifting due to structural changes and management measures such as a professional milking routine and milking hygiene [1,2,3]. While most clinical mastitis cases were once caused by cow-associated pathogens like *Staphylococcus (S.) aureus* and *Streptococcus (S.) agalactiae* [4], current studies show that the pathogens with the highest prevalence on German dairy farms originate from the environment [5,6]. Of these pathogens, *E. coli* in particular is ascribed the cases with the most severe progression [7]. Severe mastitis is commonly known as “coli mastitis” by veterinarians and farmers and feverish clinical cases are virtually equated with *E. coli* infections or Gram-negative pathogens in general. Nevertheless, in severe cases, other pathogens are also regularly detected microbiologically in the field. Moreover, *S. uberis* is a frequently detected pathogen on many German dairy farms [8], and it is likely that its frequent occurrence is sufficient to be causative even in a number of severe cases. The assumption that every severe case of mastitis is attributable to Gram-negative pathogens like *E. coli* or other coliforms may influence the fact that diagnostic methods are performed less frequently, as veterinarians and farmers supposedly consider the diagnosis to be correct solely based on the clinical picture.

Furthermore, to adapt treatment protocols to changing conditions, it is imperative to know what the chances of cure actually are outside of clinical studies and which factors influence them most significantly. The influence on cure and recurrence rate has been previously described, where mainly the effect on bacteriological cure was investigated [9,10]. An antibiotic treatment is only appropriate if a bacteriological cure can be achieved, which is defined as the elimination of the mastitis-causing pathogens from the mammary gland. Existing treatment protocols primarily focus on bacteriological cure, as clinical and cytological cure are seen as a consequence of this. A quarter is considered cytologically cured when there is no longer a high somatic cell count (SCC) as a sign of a subsided inflammatory reaction. Nonetheless, even if high bacteriological cure rates were achieved for several mastitis-causing pathogen groups, the authors usually described an inadequate overall cure rate due to a persistently elevated SCC [11]. Therefore, especially the influence of different factors on the complete cure rate is of particular interest, not only to eliminate the pathogens but also to reduce the inflammation of the udder tissue.

The purpose of the present study was to characterize occurring clinical mastitis cases as well as to describe factors influencing cure rates and recurrent cases based on the analyzation of a large number of clinical mastitis cases in Northern Germany. With the provided data, more accurate and appropriate decisions for diagnostics and treatment can be made on a daily basis, as low cure rates indicate insufficient or ineffective therapies. Raising awareness of poor chances of recovery and related recurrent cases can be beneficial so that farmers provide chronically ill animals with an anti-inflammatory drug as part of evidence-based therapy concepts instead of a continuing antibiotic treatment.

## 2. Materials and Methods

All applicable guidelines for the care and use of animals were followed. The study was approved by the Animal Welfare Committee of Hannover University, Germany. An application for a license for animal testing was not required by the local government due to the observational character of the study. The study met the International Guiding Principles for Biomedical Research Involving Animals (1985).

### 2.1. Herds and Study Design

All participating farms (*n* = 12) were free-stall dairy farms with herd sizes between 75 and 2200 dairy cows. Family-run farms as well as larger farms with up to 20 external employees took part. Participating farms had average to high level animal health management and bulk tank milk SSC ranged from 150,000 to 300,000 cells/mL milk. Most of the farms had two milking times per day, with only one having three; two farms had automatic milking systems. Average annual milk production was between 9500 and 12,200 kg. Two dairy farmers produced in accordance with the regulations of the German organic farming association “Bioland”.

The milk samples included in this study were regular samples of clinical mastitis cases sent into the laboratory of Hannover University of Applied Sciences and Arts, Germany for cytomicrobiological testing. The condition of participation in the study for farms was that they had been sending milk samples routinely for several years. Therefore, farm staff were experienced in aseptic sampling in accordance with the guidelines of the German Veterinary Association [12], as well as being trained in mastitis severity classification, based on the definition from the International Dairy Federation [13]. Milk samples from clinical mastitis cases on 12 farms in Northern Germany in the period from 2014 to 2018 were enrolled in this study. Each farm was monitored on average for half a year. In cases of clinical mastitis, post-treatment quarter samples were taken after 14 (+/−3) and after 21 (+/−3) days by veterinarians in the working group with prior consent of the farmers. The treatment was performed in accordance with the guidelines of the German Veterinary Association [14]. Standard treatment protocols provided local antibiotic treatment (intramammary treatment) for mild and moderate cases and systemic antibiotics only for severe cases as a rule. Administered pharmaceutical products differed between the farms, but they showed comparable withdrawal times.

The mastitis severity score (MS) was defined as follows: MS 1 if there was only change in the appearance of the milk (color, viscosity, consistency), MS 2 in the case of additional local clinical signs of the udder (swelling, heat), and MS 3 for cows with general clinical signs (fever, lack of appetite).

Objective variables were bacteriological cure, cytological cure, full cure, and recurrence rate at quarter level.

### 2.2. Laboratory Procedures

Milk samples were collected in test tubes containing the preserving agent boric acid (Ly20) [15]. Conventional cytomicrobiological diagnostic examinations were performed at the laboratory of Hannover University of Applied Sciences and Arts, Germany in accordance with the guidelines of the German Veterinary Association [12], which are similar to the National Mastitis Council recommendations [4]. Then, 10 μL of each well-mixed milk sample was plated with a sterile calibrated loop on a quadrant of an aesculin blood agar plate (Thermo Fisher Scientific, Langenselbold, Germany). Plates were incubated for at least 48 h at 37 °C under aerobic conditions. Isolates were Gram stained to assist in organism identification. Furthermore, morphology of colonies, aesculin hydrolysis, catalase reactivity (3 per cent H2O2; Merck, Darmstadt, Germany), and hemolysis patterns were used for identification. Gram-positive and catalase-positive cocci were identified as staphylococci. For differentiation of *S. aureus*, a clumping factor test was performed (Staph Plus Kit, DiaMondiaL, Vienna, Austria). Other staphylococci were referred to as non-*aureus* staphylococci (N*a*S). Gram-positive and catalase-negative cocci were identified as streptococci. For differentiation of aesculin hydrolyzing cocci, modified Rambach agar was used. β-d-Galactosidase-positive and aesculin hydrolyzing cocci were identified as *S. uberis*. Aesculin hydrolyzing, β-d-galactosidase-negative cocci were identified as enterococci. ß-hemolytic streptococci were characterized by Lancefield serotyping (DiaMondiaL Streptococcal Extraction Kit Sekisui Virotech, Germany). Streptococci from group C were referred to as *Streptococcus (S.) dysgalactiae*, from group B as *S. agalactiae*. Gram-positive, ß-hemolytic, catalase-negative irregular rods with V- or Y-shaped configurations were identified as *Trueperella (T.) pyogenes*. Gram-positive, catalase-positive, asporogenic colonies on aesculin blood agar were identified as coryneform bacteria. *Bacillus* species form colonies on aesculin blood agar which are catalase-positive and appear as Gram-positive rods forming endospores. Gram-negative and cytochrome oxidase-negative (Bactident oxidase, Merck, Darmstadt, Germany) rods were further differentiated using Chromocult Coliform Agar (Merck, Darmstadt, Germany). After incubation at 37 °C for 24 h, *E. coli* forms blue colonies and other coliforms form pink-red colonies. Gram-negative rods showing no mobility during the performance of the oxidative fermentative test were identified as *Klebsiella* species. Gram-negative, catalase-positive, and cytochrome oxidase-positive rod-shaped bacteria showing oxidative glucose degradation were identified as *Pseudomonas* species. Yeasts, molds, and *Prototheca* species were differentiated microscopically. Environment-associated, mastitis-causing microorganisms (*S. uberis*, *E. coli*, N*a*S, *Klebsiella* species, coliform bacteria, yeasts, *Pseudomonas* species, and *Prototheca* species) were recorded as a microbiologically positive result if at least ≥5 cfu/0.01 mL were cultured. Based on the recommendations of the National Mastitis Council, samples with two identified pathogens are covered by the definition of a mixed infection, whereas samples with more than two pathogens are described as contaminated, except in the event that a colony of a cow-associated microorganism (*S. aureus*, *S. agalactiae*, *S. dysgalactiae* or *T. pyogenes*) was found. Somascope Smart (Delta Instruments, The Netherlands) was used to determine the SCC by flow cytometry.

### 2.3. Definition of the Outcome Variables

Bacteriological cure was defined as an absence of the mastitis-causing pathogen in both post-treatment samples. If one post-treatment sample was contaminated, the other one was used to determine the bacteriological cure. Cytological cure was defined as the SCC of both post-treatment samples being less than or equal to 200,000 cells/mL milk. A case was fully cured if there was a bacteriological cure and a cytological cure concurrently (in the case of no bacterial finding, a cytological cure was taken to be a full cure). A recurrent case was present if a new clinical case was detected up to 90 days after the preceding infection in the same udder quarter.

### 2.4. Statistical Analysis

Data per case were collected in Microsoft Access and Microsoft Excel 2016 (Microsoft Corporation, Washington, Redmond, WA, USA). 

Due to the fact that the affected quarter within cow was the statistical unit of observation for the treatment outcome, clustering was used in the study (quarter in cow, cow in farm). All models contained cow and quarter within a cow as random effects to account for clustering within cows and repeated observations per quarter. Bacteriological cure, cytological cure, full cure, and recurrence rate were evaluated using mixed model logistic regression analysis where parity (lactation number: 1, 2, >2), days in milk (DIM; ≤100, 101–200, ≥201), MS (mild, moderate, severe), and pathogen group (*S. aureus*, *S. dysgalactiae*, *S. uberis*, N*a*S, *T. pyogenes*, coliforms, no growth, other, mixed infection, and contaminated) were included as fixed effects.

Cytological cure was categorized according to the cut-off value of 200,000 cells/mL as described above. For the statistical analysis, SPSS software (SPSS 26.0, IBM Corp., Armonk, NY, USA) was used. The full model was given by:

Logit (bacteriological cure, cytological cure, full cure, recurrence rate) = parity + DIM + MS + pathogen-group + (pathogen group x DIM) + (pathogen group x parity) + (pathogen group x MS) + herd (random) + cow (random) + quarter (random) + e.

A value of *p* < 0.05 was considered significant. The model quality was determined with the help of the Akaike information criterion. The random farm effect was not significant in the models but was kept as a design variable. 

## 3. Results

### 3.1. Descriptive Results

A total of 2883 clinical mastitis cases were enrolled in the study. Most cases of clinical mastitis were caused by environmental pathogens. The detailed pathogen distribution is listed in Table 1. *S. uberis* was the most frequently detected pathogen (581 cases, 20.2%), followed by 333 cases with coliforms (11.6%) and 172 cases of N*a*S (6.0%). In 1010 milk samples, no bacteriological growth was detected (35.0%).

Only 9.1% of all clinical mastitis cases were severe cases with general disorder of condition (249/2732), 35.7% of the detected cases had a MS2 (976/2732), and most clinical mastitis cases were classified as mild (55.2% (1507/2732)). The detailed distribution of the MS within the pathogen groups is shown in Table 2. Of all cases with *S. uberis*, 88.2% were mild or moderate and 11.8% severe. In contrast, 24.4% of all cases with proven coliform pathogens were severe cases. Of the total cases, 26.5% of all severe cases were caused by *S. uberis* due to the large number of this pathogen, and 30.5% by coliforms. For all other pathogen groups, severe clinical conditions occurred only in 4.5 to 7% of the cases, with the exception of mixed infections, where 10.9% of cases were severe.

Most clinical mastitis cases occurred in the first 100 days after calving, with two cases shortly before first calving (46.5% (1341/2883)), the number of cases decreasing as lactation progressed (101–200 DIM = 28.3% (816/2883); DIM ≥201 = 25.2% (726/2883)). The detailed distribution by phase of lactation is shown in Table 3. 

In respect of the parity, most cases occurred in animals from the third lactation onwards (55.1% (1588/2883)) (Table 4). A total of 24.8% of the cases occurred in the second lactation (716/2883) and 20.1% occurred in primiparous cows (579/2883). The pathogen distribution over the different lactations shows that *S. uberis* and N*a*S occurred relatively more frequently during first lactation than in the following lactations (*S. uberis*: 24.4 vs. 19.4 and 19%; N*a*S: 8.5 vs. 4.7 and 5.6%). In contrast, from the second lactation onwards, proportionately more cases were caused by coliform pathogens (coliforms: 7.1 vs. 12.8 and 12.6%; no growth: 27.1 vs. 36.3 and 37.3%).

### 3.2. Results of Mixed Regression Models

#### 3.2.1. Bacteriological Cure

A total of 1062 of 1448 clinical mastitis cases achieved a bacteriological cure (73.3%). The pathogen group was associated with bacteriological cure (*p* < 0.001) (Table 5), just like DIM (*p* = 0.006) and the MS (*p* = 0.02). In the following, only the significant subgroups in the tables are shown, because a complete representation of all would be too extensive. Parity did not show any significance in the multivariable model for bacteriological cure. The detailed bacteriological cure rates for the respective pathogens are shown in Table 6. Animals with mastitis caused by coliform bacteria had the highest bacteriological cure rate of 87.1%. *S. dysgalactiae* had a comparable bacteriological cure rate of 82.9%, while all other pathogen groups showed a significantly lower bacteriological cure rate with coliforms as reference. For animals with *S. aureus*, the risk of not being bacteriologically cured was seven times higher than for animals with coliform pathogens, the former having the lowest cure rate of 44.7% (*p* < 0.001).

Regarding the period of lactation, animals had greater chances of bacteriological cure at the end of lactation (DIM ≥201: *p* = 0.002). In order to further analyze this result, an interaction between bacteriological findings and period of lactation was investigated in the model. No significance for an interaction could be demonstrated. It was shown that the bacteriological cure rate increased the more severe the mastitis was (MS3: *p* = 0.016).

#### 3.2.2. Cytological Cure

The overall cytological cure rate was 22.3% (537/2405). Table 6 gives the cure rates for the individual pathogen groups. After model building, remaining explanatory variables were pathogen group (*p* < 0.001), pathogen group x DIM (*p* < 0.001), and pathogen group x parity (*p* = 0.012) (Table 7). In contrast to the bacteriological cure rate, MS had no significant influence on cytological cure.

Animals with clinical mastitis caused by N*a*S (*p* = 0.017) had a significantly higher cytological cure rate vs. cases caused by coliforms. Clinical mastitis cases with no pathogen growth had a higher risk of not achieving a cytological cure when they happened in middle or late lactation vs. in the first 100 days postpartum (DIM 101–200: *p* = 0.001; DIM ≥201: *p* < 0.001). Similarly, the cytological cure rate of *S. uberis* was worse in the middle of lactation vs. in the first 100 days (*S. uberis* ×DIM 101–200: *p* = 0.009). Cases caused by coliforms or by “other” pathogens, on the other hand, had a lower cytological cure rate in the first 100 DIM (coliforms × DIM ≥201: *p* = 0.022; others × DIM 101–200: *p* = 0.042). In terms of parity, cytological cure rates decreased significantly for cases with N*a*S during the second lactation and in the third lactation onwards (N*a*S × lactation number = 2: *p* = 0.005; N*a*S × lactation number >2: *p* = 0.21) and for cases caused by coliforms or with mixed infections from the third lactation onwards vs. cases from primiparous animals (coliforms × lactation number >2: *p* = 0.27; N*a*S × lactation number >2: *p* = 0.01).

#### 3.2.3. Full Cure

The overall proportion of full cure was 21.4% (514/2405). The full cure rates for pathogen groups are presented in Table 6. In the final mixed model, significant variables were the pathogen group (*p* < 0.001) and pathogen group × DIM (*p* = 0.001). Parity, MS, and DIM without interactions did not influence the full cure rate significantly (Table 8).

Clinical mastitis cases without pathogen detection had a significantly higher chance of full cure (*p* < 0.001), whereas it was shown that especially no growth cases in the first 100 DIM were almost twice as likely to achieve a full cure than cases in the middle or late lactation (no growth × DIM 101–200: *p* = 0.001; no growth × DIM ≥201: *p* < 0.001). A similar trend was shown for cases caused by *S. uberis*, happening between 101 and 200 DIM, with less chance of full cure than at the beginning of lactation (*S. uberis* × DIM 101–200: *p* = 0.006). In contrast, clinical mastitis cases with coliform bacteria had a higher full cure rate during late lactation (coliforms × DIM ≥201: *p* = 0.23).

#### 3.2.4. Recurrent Cases

In the 90-day follow-up period, 18.8% of the mastitic udder quarters experienced a recurrent case (508/2702) (Table 6). Significant variables were pathogen group (*p* < 0.001), parity (*p* = 0.001), DIM (*p* = 0.002), and MS (0.008) (Table 9).

Cases caused by *S. uberis* showed a significantly higher risk for recurrent intramammary infections (*p* = 0.028), whereas cases with N*a*S had significantly less recurrent cases vs. coliforms (*p* = 0.004). Cows in their third or higher lactation had a higher risk of developing recurrent mastitis than primiparous cows (lactation number >2: *p* < 0.001). Moreover, the risk of a recurrent case decreased at the end of lactation (DIM >201: *p* = 0.001) and if animals had moderate or severe mastitis (MS2: *p* = 0.01; MS3: *p* = 0.014).

## 4. Discussion

To the best of our knowledge, this is the largest field study ever conducted on cure rates of clinical mastitis in Europe. The aim of this study was to quantify treatment success of current standard treatment protocols and to characterize clinical mastitis cases in Germany. Due to the aforementioned objectives of the study, we were dependent on the cooperation of the participating dairy farms. All of these farms had been sending milk samples routinely for several years to Hannover University of Applied Sciences and Arts, Germany. This provided good sample quality and ensured that all cases were recorded. Furthermore, this also implies that the farms tended to be well managed in general and their standard operating procedures in the udder health sector were more professional and qualified with routine diagnostics than the national average. This may have influenced the results of our study.

### 4.1. Characterization

*Streptococcus uberis* was the most frequently detected pathogen in this study, accounting for 20.2% of all cases. This corresponds to the results of other recent studies on the pathogen prevalence of clinical mastitis [16,17]. It is described that in herds where transmission of contagious mastitis pathogens is controlled through the implementation of mastitis prevention programs for the milking process, mastitis-causing pathogens originate from the animals’ surrounding environment [18]. A further indication that this also applies to the farms included in our study is the low proportion of 3.7% of proven cases caused by *S. aureus*. In order to develop an effective prevention program against environmental pathogens, it must be considered that a cow-associated contagious form of some *S. uberis* strains has been previously described [19]. Wente et al. [8] compared *S. uberis* strains from clinical cases and the environment at farm level. Isolates were detected in liners, with one matching a mastitis milk strain, which suggests that bacterial transmission takes place during milking. Moreover, a limited variety of *S. uberis* mastitis strains on one farm points to the fact that its transmission is cow-associated. For farms with *S. uberis* as the dominant pathogen, the authors recommend comparing mastitis strains by means of pulsed-field gel electrophoresis to obtain a better understanding of transmission pathways.

To our knowledge, no previous study has been conducted in Germany characterizing clinical mastitis cases, classifying them according to their severity. Even so, in the present study, nearly 90% of the cases caused by *S. uberis* were non-severe, and almost 30% of all severe cases were caused by *S. uberis* due to its high frequency of occurrence. Coliform bacteria were the most common pathogen group detected in severe cases. This is consistent with a study from the USA on the severity of clinical mastitis cases in 2013 [20]. Nevertheless, the overall proportion of coliform pathogens in our study differs from their results, as the earlier study detected coliform pathogens most frequently in large American herds, regardless of severity. However, when analyzing the distribution of clinical cases in terms of the severity levels within the coliform pathogen group, coliform bacteria were much more likely to cause non-severe cases (75.6%) than severe cases (24.4%). A similar distribution pattern was reported by Hogan et al. [21]. For a strategically prudent antibiotic treatment, two important factors can be deduced from the results on MS. The first is that in Northern Germany, in a severe case, it is equally likely that a Gram-positive or a Gram-negative pathogen is present. Even if in feverish cases, a parenterally administered broad-spectrum antibiotic is recommended to treat a possible bacteremia [22], the causative agent should still be identified or a classification according to Gram behavior using on-farm culture should be considered. This enables local antibiotic treatment adapted to the pathogen and, if appropriate, the change to a narrow spectrum antibiotic. The second is that Gram-negative pathogens tend to cause non-severe cases. Veterinarians and farmers must be aware that no antimicrobial treatment is necessary in such cases and that by performing a rapid test, they can reduce their antibiotic usage without negative effects [23,24,25,26]. 

Currently, the resistance situation of mastitis-causing pathogens in Germany is not considered critical [27]. Gram-positive mastitis pathogens, especially streptococci and staphylococci, showed high in-vitro sensitivity to penicillins in a study from 2020, which are therefore considered the preferred choice in these cases [28]. In order to prevent the risk of further development of resistance, the European guidelines for the prudent use of antimicrobials in veterinary medicine stipulate that a narrow-spectrum antibiotic should be used if possible [29]. These recommendations must be implemented in prospective treatment concepts. An on-farm culture that reliably detects Gram-positive pathogens can give farmers the ability to specifically apply narrow-spectrum antibiotics in these cases. Considering the distribution of pathogens between the parity numbers, N*a*S and streptococci occurred particularly frequently in primiparous cows, as already described in previous studies [6,30]. 

The most clinical mastitis cases occurred in the first 100 days after calving and decreased as lactation progressed. This result has already been described in detail, as cows have a weakened immune system in the period around calving due to an energy deficit caused by the start of milk production [31,32]. To lastingly improve the cows’ start in lactation, different management factors like using an internal teat sealant, a selective dry cow therapy practice, and a high hygiene standard during the dry period and in the calving area were associated with a decreased new infection rate after calving [33,34].

### 4.2. Results of Mixed Regression Models

Overall, the bacteriological cure rate was high, with 73.3%. The pathogen group had the greatest influence, with the highest bacteriological cure rates for cases caused by coliforms or *S. dysgalactiae* being over 82% and the lowest for cases caused by *S. aureus* being 44.7%. In clinical treatment trials, bacteriological cure rates of clinical mastitis caused by *S. aureus* of less than 30% to approximately 50% have been achieved [35,36]. Thus, the bacteriological cure rate achieved in our field study is comparatively high even for *S. aureus* cases. While a similar study of naturally occurring clinical mastitis in the U.S. categorized the pathogen groups differently in its presented results [20], the bacteriological cure rates in our study tended to be higher for both Gram-positive and Gram-negative pathogens. The number of DIM had a significant impact on bacteriological cure, with cases after more than 200 DIM having a higher bacteriological cure rate. This is contrary to the findings of McDougall et al. [37], who demonstrated a decreasing bacteriological cure the later the mastitis occurred in lactation. However, it has also been described that the lactation stage has no effect on bacteriological cure outcomes [35]. McDougall stated in his findings that the later detected cases in lactation may have existed longer and were not treated in time. If one assumes in our study that the participating farms had a functioning animal observation in the context of a more professional health management system and thus all cases were promptly detected, a reason for the worse bacteriological cure rates at the beginning of lactation could be that the animals were affected with other metabolically caused diseases. The severity of mastitis also had an impact on bacteriological cure. The bacteriological cure rate increased with the increasing severity of the case. The study by Oliveira et al. [20] showed the same significance of the MS, but in contrast to our results, the majority of severe cases were caused by Gram-negative pathogens, which is not the case in our data set. From this, it can be concluded that even severe mastitis caused by Gram-positive pathogens have a great chance of bacteriological cure. 

The overall cytological cure rate was poor in the present study, and, as a consequence, the full cure rate was low, too. In both models, the pathogen group was the most decisive factor for the outcome. In the model for cytological cure, cases caused by N*a*S had the highest cytological cure, with 38.2%. Parity and DIM were only significant in interaction with the pathogen group. Primiparous cows achieved higher cytological cure rates than omniparous animals in cases of N*a*S, coliforms, or mixed infections. Moreover, cases caused by *S. uberis* and with no microbial growth had a greater chance of cytological cure at the beginning of lactation. The overall bacteriological cure rate was 73.3%, but the overall cytological cure rate was only 22.3%. From these results, it can be deduced that a bacteriological cure alone is not sufficient to reduce the inflammation in the affected udder quarter. Since the SCC is probably the most important target variable from the farmers’ point of view, more research is needed to reduce inflammation.

In the full cure model, the pathogen group and DIM in interaction with the pathogen group again had a significant impact on the outcome. Cases with no bacterial growth had a significantly higher full cure rate, with 31.6%. These no growth cases and also cases caused by *S. uberis* had an even greater chance of full cure in the first 100 days of lactation. Considering all cure rates, it can be stated that the highest bacteriological cure can be achieved in the first 100 days of lactation, regardless of the pathogen. For certain pathogens, including *S. uberis*, even full cure rate is significantly better at the beginning of lactation, irrespective of the MS. Therefore, especially cases at the beginning of lactation should be treated appropriately, including all mild cases with Gram-positive pathogens like *S. uberis* [25].

The overall recurrence rate was 18.8% is this study. This coincides with results of Oliveira et al. [20], with 21.4% of all clinical mastitis cases experiencing a recurrent clinical mastitis. Cases caused by *S. uberis* showed a higher risk of developing recurrent intramammary infections, whereas cases with N*a*S had significantly fewer recurrent cases. According to a recent study, *S. uberis* is more likely to cause recurrences than other species, although these are very often not the result of persistent infections [38]. Zadoks et al. [39] previously described that quarters, recovered from an infection with *S. uberis*, showed an increased recurrence rate. Our results support the hypothesis that a previous infection does not provide immunological protection against subsequent infections but rather makes the udder tissue susceptible to further infections.

Cows in their third or higher lactation had a higher risk of developing a recurrent clinical mastitis than primiparous cows. A former study showed a tendency for the proportion of recurrent cases to increase with parity [40]. The risk of a recurrent case decreased if animals had moderate or severe mastitis. On the contrary, Oliveira et al. [20] could not find any associations between the MS and recurrent clinical mastitis. This result may have been influenced by cows with severe mastitis leaving the herds, which was not recorded in the study. The finding that the risk of a recurrence at the end of lactation is reduced is explained by the fact that we only recorded recurrences during the current lactation in this study.

Treatment was recorded as a random factor across the herds but never had a significant effect on target variables. The treatment concepts were similar between the farms, but the antibiotic products were different. This is an indication that the antibiotics available on the market are effective. Furthermore, it suggests that, with our administered treatment, we had less influence on the outcome than we would have liked to.

As the aim of existing treatment protocols is to achieve a bacteriological cure of the infected udder quarters, veterinarians and farmers focus primarily on antibiotic treatment. The present study demonstrated that most animals were bacteriologically cured with standard treatment protocols, but not fully cured. As an increased SCC leans to a higher risk of new infections and milk loss, the treatment should focus on supportive treatment in order to decrease the inflammation of the udder tissue.

## 5. Conclusions

Environmental pathogens are the major cause of clinical mastitis on dairy farms in Northern Germany, with 20.2% of all cases attributable to *S. uberis*. Regarding the pathogen distribution of severe mastitis, coliform bacteria were detected in 30.5% of the cases and *S. uberis* was detected almost as frequently, namely in 26.5% of the cases. This indicates that even severe cases are often caused by Gram-positive pathogens. Thus, identifying the pathogen group is necessary in order to adjust the choice of antimicrobial agents. This enables prudent use of antibiotics. Of the factors analyzed, the causative pathogen had the greatest influence on cure rates and recurrent cases. Overall, the bacteriological cure rate was high, but the full cure rate was low, namely 21.4%. From these results, it can be concluded that there is no need for new antibiotic therapies but, rather, treatment concepts that alleviate the inflammatory reaction.

## Figures and Tables

**Table 1:** Detailed microbiological results based on conventional diagnostic methods (*n* = 2883 mastitic udder milk samples).

Microbiological Findings	*n*	%
*Streptococcus uberis*	581	20.2
N*a*S	172	6.0
*Staphylococcus aureus*	164	3.7
*Streptococcus dysgalactiae*	86	3.0
*Trueperella pyogens*	38	1.3
Coliforms	333	11.6
Mixed infections	106	3.7
Others ^1^	193	6.7
No growth	1010	35.0
Contaminated ^2^	200	6.9
Total	2883	100

^1^: *Prototheca* spp., *Bacillus* spp., *Enterococcus* spp., yeast, *Pseudomonas* spp., *Corynebacterium* spp., other streptococci. ^2^: More than two different pathogens were detected in one sample. N*a*S: Non-*aureus* staphylococci.

**Table 2:** Microbiological results based on conventional diagnostic methods (*n* = 2732 mastitic udder milk samples) and their distribution by mastitis severity score (MS).

	Mastitis Severity Score									
	Mild (MS1)			Moderate (MS2)			Severe (MS3)			
Microbiological Findings	*n*	% ^1^	% ^2^	*n*	% ^1^	% ^2^	*n*	% ^1^	% ^2^	Total ^3^ (*n*)
*Streptococcus uberis*	243	43.4	16.1	251	44.8	25.7	66	11.8	26.5	560
N*a*S	108	67.1	7.2	42	26.1	4.3	11	6.8	4.4	161
*Staphylococcus aureus*	93	59.6	6.2	56	35.9	5.7	7	4.5	2.8	156
*Streptococcus dysgalactiae*	44	55.0	2.9	31	38.8	3.2	5	6.3	2.0	80
*Trueperella pyogens*	14	38.9	0.9	20	55.6	2.0	2	5.6	0.8	36
Coliforms	102	32.8	6.8	133	42.8	13.6	76	24.4	30.5	311
Mixed infections	49	48.5	3.3	41	40.6	4.2	11	10.9	4.4	101
Others ^4^	111	60.0	7.4	61	33.0	6.3	13	7.0	5.2	185
No growth	628	66.1	41.7	273	28.7	28.0	49	5.2	19.7	950
Contaminated ^5^	115		7.6	68		7.0	9		3.6	192
Total	1507 ^3^		100	976 ^3^		100	249 ^3^		100	2732 ^6^

^1^: Proportion of cases of all cases caused by the pathogen group. ^2^: Proportion of cases of all cases of the respective MS. ^3^: Number of cases per pathogen group with MS. ^4^: *Prototheca* spp., *Bacillus* spp., *Enterococcus* spp., yeast, *Pseudomonas* spp., *Corynebacterium* spp., other streptococci. ^5^: More than two different pathogens were detected in one sample. ^6^: Number of cases with recorded MS. MS: mastitis severity score, N*a*S: non-*aureus* staphylococci.

**Table 3:** Microbiological results based on conventional diagnostic methods (*n* = 2883 mastitic udder milk samples) and their distribution by phase of lactation.

	Phase of Lactation									
	Early Lactation (DIM ≤ 100)			Mid Lactation (DIM 101–200)			Late Lactation (DIM ≥ 201)			
Microbiological Findings	*n*	% ^1^	% ^2^	*n*	% ^1^	% ^2^	*n*	% ^1^	% ^2^	Total (*n*)
*Streptococcus uberis*	278	47.8	20.7	169	29.1	20.7	134	23.1	18.5	581
N*a*S	97	56.4	7.2	43	25.0	5.3	32	18.6	4.4	172
*Staphylococcus aureus*	66	40.2	4.9	43	26.2	5.3	55	33.5	7.6	164
*Streptococcus dysgalactiae*	43	50.0	3.2	23	26.7	2.8	20	23.3	2.8	86
*Trueperella pyogens*	21	55.3	1.6	6	17.8	0.7	11	28.9	1.5	38
Coliforms	161	48.3	12.0	97	29.1	11.9	75	22.5	10.3	333
Mixed infections	63	59.4	4.8	20	18.9	2.5	23	21.7	3.2	106
Others ^3^	94	48.7	7.0	46	33.2	5.6	53	27.5	7.3	193
No growth	447	44.3	33.3	289	28.6	35.4	274	27.1	37.7	1010
Contaminated ^4^	71		5.3	80		9.8	49		6.7	200
Total	1341			816			726			2883

^1^: Proportion of cases of all cases caused by the pathogen group. ^2^: Proportion of cases of all cases of the respective phase of lactation. ^3^: *Prototheca* spp., *Bacillus* spp., *Enterococcus* spp., yeast, *Pseudomonas* spp., *Corynebacterium* spp., other streptococci. ^4^: More than two different pathogens were detected in one sample. DIM: days in milk, N*a*S: non-*aureus* staphylococci.

**Table 4:** Microbiological results based on conventional diagnostic methods (*n* = 2883 mastitic udder milk samples) and their distribution by lactation number.

	Lactation									
	First Lactation			Second Lactation			Third Lactation and Beyond			
Microbiological Findings	*n*	% ^1^	% ^2^	*n*	% ^1^	% ^2^	*n*	% ^1^	% ^2^	Total (*n*)
*Streptococcus uberis*	141	24.3	24.4	139	23.9	19.4	301	51.8	19.0	581
N*a*S	49	28.5	8.5	34	19.8	4.7	89	51.7	5.6	172
*Staphylococcus aureus*	51	31.1	8.8	40	24.4	5.6	73	44.5	4.6	164
*Streptococcus dysgalactiae*	25	29.1	4.3	17	19.8	2.4	44	51.2	2.8	86
*Trueperella pyogens*	6	15.8	1.0	11	28.9	1.5	21	55.3	1.3	38
Coliforms	41	12.3	7.1	92	27.6	12.8	200	60.1	12.6	333
Mixed infections	33	31.1	5.7	23	21.7	3.2	50	47.2	3.1	106
Others ^3^	38	19.7	6.6	51	26.4	7.1	104	53.9	6.5	193
No growth	157	15.5	27.1	260	25.7	36.3	593	58.7	37.3	1010
Contaminated ^4^	38		6.6	49		6.8	113		7.1	200
Total	579			716			1588			2883

^1^: Proportion of cases of all cases caused by the pathogen group. ^2^: Proportion of cases of all cases of the respective phase of lactation. ^3^: *Prototheca* spp., *Bacillus* spp., *Enterococcus* spp., yeast, *Pseudomonas* spp., *Corynebacterium* spp., other streptococci. ^4^: More than two different pathogens were detected in one sample. N*a*S: non-*aureus* staphylococci.

**Table 5:** Final mixed logistic regression model for bacteriological cure of clinical mastitis from 12 different herds in Northern Germany.

Effect	Coefficient	SE	t Value	*p*-Value	OR	95% CI
Pathogen group						
*S. uberis*	1.01	0.22	4.55	<0.001	2.73	1.77–4.21
N*a*S	0.88	0.29	3.08	0.002	2.41	1.38–4.22
*S. aureus*	1.98	0.27	7.39	<0.001	7.27	4.29–12.29
*T. pyogenes*	1.44	0.43	3.33	0.001	4.21	1.80–9.83
Coliforms				Reference		
Mixed infection	1.63	0.30	5.51	<0.001	5.11	2.86–9.13
Others ^1^	0.71	0.27	2.59	0.01	2.02	1.19–3.45
DIM						
≥201	−0.57	0.18	−3.17	0.002	0.57	0.40–0.81
≤100				Reference		
Mastitis score						
MS3	−0.60	0.25	−2.41	0.016	0.55	0.34–0.90
MS2	−0.30	0.14	−2.10	0.036	0.74	0.56–0.98
MS1				Reference		

^1^: *Prototheca* spp., *Bacillus* spp., *Enterococcus* spp., yeast, *Pseudomonas* spp., *Corynebacterium* spp., other streptococci. N*a*S: non-*aureus* staphylococci, DIM: days in milk, MS: mastitis severity score.

**Table 6:** Outcomes by microbiological findings of clinical mastitis cases (*n* = 2883) in Northern Germany.

	Bacteriological Cure		Cytological Cure		Full Cure		Recurrent Cases	
Microbiological Findings	*n*	%	*n*	%	*n*	%	*n*	%
*Streptococcus uberis*	376/509	73.9 ^a^	51/483	10.5	50/483	10.4	115/529	21.7 ^a^
N*a*S	117/152	77.0 ^a^	55/144	38.2 ^a^	40/144	27.8	8/163	4.9 ^a^
*Staphylococcus aureus*	63/141	44.7 ^a^	21/140	15.0	19/140	13.6	42/158	26.6
*Streptococcus dysgalactiae*	63/76	82.9	17/76	22.4	17/76	22.4	19/82	23.2
*Trueperella pyogens*	18/31	58.1 ^a^	5/30	16.7	5/30	16.7	7/32	21.9
Coliforms	243/279	87.1 ^r^	39/256	15.2 ^r^	38/256	14.8 ^r^	53/317	16.7 ^r^
Mixed infections	59/97	60.8 ^a^	16/93	17.2	14/93	15.1	22/100	22.0
Others ^1^	121/161	75.2 ^a^	24/155	15.5	22/155	14.2	37/176	21.0
No growth			271/857	31.6	271/857	31.6 ^a^	148/950	15.6
Contaminated ^2^			38/171	22.2	38/171	22.2	55/132	29.4
Total	1060/1446	73.3	537/2405	22.3	514/2405	21.4	508/2702	18.8

^a^: pathogen (group) shows significance in mixed model. ^r^: reference. ^1^: *Prototheca* spp., *Bacillus* spp., *Enterococcus* spp., yeast, *Pseudomonas* spp., *Corynebacterium* spp., other streptococci. ^2^: More than two different pathogens were detected in one sample. N*a*S: non-*aureus* staphylococci.

**Table 7:** Final mixed logistic regression model for cytological cure of clinical mastitis from 12 different herds in Northern Germany.

Effect	Coefficient	SE	t Value	*p*-Value	OR	95% CI
Pathogen group						
N*a*S	−1.28	0.54	−2.39	0.017	0.28	0.10–0.79
Coliforms				Reference		
Pathogen group × DIM						
*S. uberis* × DIM 101–200	1.15	0.44	2.61	0.009	3.16	1.33–7.47
*S. uberis* × DIM ≤100				Reference		
Coliforms × DIM ≥201	−0.95	0.42	−2.29	0.022	0.39	0.17–0.87
Coliforms × DIM ≤100				Reference		
Others ^1^ × DIM 100–200	−0.57	0.18	−3.17	0.002	0.57	0.40–0.81
Others × DIM ≤100				Reference		
No growth × DIM ≥201	0.78	0.20	3.90	<0.001	2.18	1.47–3.22
No growth × DIM 101–200	0.62	0.19	3.35	0.001	1.86	1.29–2.67
No growth × DIM ≤100				Reference		
Pathogen group × parity						
N*a*S × lactation number >2	0.96	0.41	2.31	0.021	2.60	1.16–5.85
N*a*S × lactation number =2	1.71	0.61	2.79	0.005	5.50	1.66–18.25
N*a*S × lactation number =1				Reference		
Coliforms × lactation number >2	1.07	0.49	2.21	0.027	2.93	1.13–7.58
Coliforms × lactation number =1				Reference		
Mixed infection × lactation number >2	2.93	1.13	2.59	0.01	18.68	2.04–171.15
Mixed infection × lactation number =1				Reference		

^1^: *Prototheca* spp., *Bacillus* spp., *Enterococcus* spp., yeast, *Pseudomonas* spp., *Corynebacterium* spp., other streptococci. N*a*S: non-*aureus* staphylococci, DIM: days in milk.

**Table 8:** Final mixed logistic regression model for full cure of clinical mastitis from 12 different herds in Northern Germany.

Effect	Coefficient	SE	t Value	*p*-Value	OR	95% CI
Pathogen group						
No growth	−1.22	0.29	−4.26	<0.001	0.30	0.17–0.52
Coliforms				Reference		
Pathogen group × DIM						
*S. uberis* × DIM 101–200	1.20	0.44	2.74	0.006	3.31	1.40–7.82
*S. uberis* × DIM ≤100				Reference		
Coliforms × DIM ≥201	−0.93	0.41	−2.28	0.023	0.39	0.18–0.88
Coliforms × DIM ≤100				Reference		
No growth × DIM ≥201	0.78	0.20	3.95	<0.001	2.19	1.48–3.23
No growth × DIM 101–200	0.63	0.18	3.41	0.001	1.87	1.31–2.69
No growth × DIM ≤100				Reference		

DIM: days in milk.

**Table 9:** Final mixed logistic regression model for recurrent cases of clinical mastitis from 12 different herds in Northern Germany.

Effect	Coefficient	SE	t Value	*p*-Value	OR	95% CI
Pathogen group						
*S. uberis*	0.45	0.20	2.20	0.028	1.57	1.05–2.34
N*a*S	−1.16	0.41	−2.85	0.004	0.31	0.14–0.70
Coliforms				Reference		
Parity						
>2	0.55	0.16	3.54	<0.001	1.73	1.28–2.35
2	0.33	0.17	1.91	0.056	1.39	0.99–1.95
1						
DIM						
≥201	−0.49	0.15	−3.33	0.001	0.62	0.46–0.82
≤100				Reference		
Mastitis score						
MS3	−0.53	0.22	−2.45	0.014	0.59	0.38–0.90
MS2	−0.31	0.12	−2.58	0.01	0.73	0.58–0.93
MS1				Reference		

N*a*S: non-*aureus* staphylococci, DIM: days in milk, MS: mastitis severity score.
